# High prevalence of toxic shock syndrome toxin–producing epidemic methicillin-resistant *Staphylococcus aureus* 15 (EMRSA-15) strains in Kuwait hospitals

**DOI:** 10.1016/j.nmni.2016.03.008

**Published:** 2016-04-01

**Authors:** E.E. Udo, S.S. Boswihi, N. Al-Sweih

**Affiliations:** Department of Microbiology, Faculty of Medicine, Kuwait University, Kuwait

**Keywords:** DNA microarray, EMRSA-15, MLST, MRSA, SCC*mec* typing, spa typing

## Abstract

This study characterized EMRSA-15 isolates obtained from patients in Kuwait hospitals for their genotypic relatedness, antibiotic resistance and carriage of virulence genes using pulsed-field gel electrophoresis (PFGE), coagulase serotyping, SCC*mec* subtyping, spa typing, multilocus sequence typing and DNA microarray. The isolates were resistant to trimethoprim (75.6%), ciprofloxacin (29.7%), erythromycin and clindamycin (24.3%), tetracycline (19.0%), and gentamicin and kanamycin (21.6%). All 37 isolates belonged to sequence type (ST) 22, coagulase type XI, three PFGE types and eight subtypes, ten spa types including t223 (51.3%), t852 (13.5%), t032 (8.1%), t790 (8.1%), t3107 (5.4%) and one each of t309, t2251, t3935, t5708 and t5983. Twenty-six isolates (70.2%) carried SCC*mec* IVa, eight isolates carried SCC*mec* IV and three isolates carried SCC*mec* IVh. All isolates carried *agr1, cap5* and egc gene cluster (*seg, sei, selm, seln, selo,* and *selu*). *tst* (toxic shock syndrome toxin) was detected in 23 isolates. Eight isolates (21.6%) were positive for Panton-Valentine leukocidin (PVL). Genotypic analysis revealed that 62.1% of the isolates comprising ST22-IVa-t223 (51.3%) and ST22-IVa-t309/t2251/t3935/t5708 (10.8%) were CC22-[tst1^+^] UK EMRSA-15/Middle Eastern variant, 21.6% were CC22-PVL^+^ EMRSA-15 variant and 16.2% were CC22-UK EMRSA-15/Barnim clone. These results show that the *tst*1 positive-ST22-IVa-t223 (Middle Eastern variant) and the CC22-PVL^+^ EMRSA-15 variant were the dominant EMRSA-15 variants in Kuwait hospitals.

## Introduction

Methicillin-resistant *Staphylococcus aureus* (MRSA) strains continue to be isolated from both healthcare- and community-associated infections in different parts of the world. The increase in the number of MRSA infections reported worldwide has been accompanied by changes in the characteristics of MRSA strains emerging in different parts of the world [1]. Consequently, epidemiologic typing using a combination of phenotypic and genotypic typing methods has contributed to our understanding of changes in the clonal distribution of MRSA isolated in different geographical locations over time. Whereas MRSA isolates obtained in the 1960s (early or classic MRSA) were usually susceptible to majority of non-β-lactam antibiotics, those isolated in the late 1970s and beyond were multiply resistant to non-β-lactam antibiotics and were described as epidemic MRSA (EMRSA) because of their capacity to spread extensively and cause serious infections among hospitalized patients [1], [2]. Epidemiologic typing by Kerr *et al*. [3] and Aucken *et al*. [4] identified 17 different EMRSA clones, designated EMRSA-1 to EMRSA-17, in the United Kingdom.

The EMRSA-15 clone was first characterized in England on the basis of phage typing (a weak lysis pattern with phage 75), susceptibility to antimicrobial agents and failure to produce urease [5]. The urease-negative phenotype resulted from a single nucleotide deletion within the *UreC* gene, leading to a frameshift inactivation of the alpha subunit of the urease gene [6]. Subsequently, multilocus sequence typing of the EMRSA-15 strains classified them as multilocus sequence (ST) type 22 (ST22) [7].

EMRSA-15 strains are an important cause of nosocomial bacteraemia in many UK and Irish hospitals [8], and they are a major pathogen in other European healthcare facilities [9], [10]. EMRSA-15 strains have also been isolated in Australia [11], Singapore [12], India [13], [14], Malaysia [15], Kuwait [16], Saudi Arabia [17], United Arab Emirates [18], Qatar [19] and Oman [20]. The survival and widespread transmission of EMRSA-15 strains have been attributed to genetic changes within the bacterial genome due to the acquisition of antibiotic resistance and virulence genes and adaptation to the healthcare environment [6].

Molecular characterization of EMRSA-15 strains isolated in the Gulf Cooperative Council (GCC) countries have revealed different genetic backgrounds with different antibiotic susceptibility patterns. Whereas EMRSA-15 isolates from the United Arab Emirates belonged to spa types t032 or t005 [18], the majority of recent isolates from Qatar [19] and Oman [20] belong to t852.

In this study, we investigated EMRSA-15 isolates obtained in Kuwait public hospitals using a combination of molecular typing methods to establish their genetic relatedness to EMRSA-15 isolated in the United Kingdom and other GCC countries.

## Materials and Methods

### MRSA isolates

A total of 37 EMRSA-15 strains, isolated in the years 2005 (four isolates representing four pulsed-field gel electrophoresis (PFGE) patterns) and 2010 (33 isolates), were included in this study. These MRSA isolates were among clinical isolates submitted to the MRSA Reference Laboratory, Kuwait, for molecular typing. The MRSA isolates were obtained as part of routine bacteriology services in the individual hospital laboratories and were archived at the MRSA Reference Laboratory, Kuwait. The MRSA isolates were isolated at ten hospitals: Mubarak (nine isolates), Al Sabah (seven isolates), Ibn Sina (five isolates), maternity hospital (five isolates), Adan (three isolates), Al Razi (three isolates), Farwaniya (two isolates), Jahra (one isolates), Armed Forces (one isolate) and Chest Disease Hospital (one isolates). Isolates were identified by cultural characteristics, Gram staining, and positive tube coagulase and DNase tests. The isolates were preserved in glycerol 40% (v/v) in brain heart infusion broth (Oxoid, Basingstoke, UK) at −80°C. They were recovered by subculturing in brain–heart infusion broth at 37°C for 24 hours followed by two further subcultures on brain–heart infusion agar. Preliminary identification of MRSA isolates as EMRSA-15 was based on negative urease test [5] and carriage of SCC*mec* type IV genetic element.

### Urease production

Urease production was detected on Christensen urea agar slope after 72 hours' incubation at 35°C as described previously [5], [16].

### Antibacterial susceptibility testing

Antibacterial susceptibility testing was performed by the disk diffusion method [21] with the following antimicrobial disks (Oxoid): benzyl penicillin (10 U), cefoxitin (30 μg), kanamycin (30 μg), mupirocin (200 and 5 μg), gentamicin (10 μg), erythromycin (15 μg), clindamycin (2 μg), chloramphenicol (30 μg), tetracycline (10 μg), trimethoprim (2.5 μg), fusidic acid (10 μg), rifampicin (5 μg), ciprofloxacin (5 μg), teicoplanin (30 μg) and linezolid (30 μg). Minimum inhibitory concentration for cefoxitin, vancomycin and teicoplanin were determined with Etest strips (bioMérieux, Marcy l’Étoile, France) according to the manufacturer's instructions. *S. aureus* strain ATCC 25923 was used as a quality control strain for susceptibility testing. The Dtest was used to test for inducible resistance to clindamycin.

### Molecular typing of isolates

Isolates were typed by PFGE, coagulase gene typing, SCC*mec* typing, spa typing and multilocus sequence typing (MLST). PFGE was performed on all 37 MRSA isolates as described previously [22]. DNA for PCR amplification was isolated and purified as described previously [20]. Coagulase typing was performed as described previously [23]. For the detection of coagulase type XI, primer pair (coaS-F) 5′-TGGGCAATTACATTTTG GAG-3′ and (coaS-XI-R) 5′-TCGTTTGGGTAAGTTGCTTT-3′ (395 bp) were designed and used in this study. PCR amplification was carried out on a Gene AMP PCR System 9700 instrument (Thermo Fisher Scientific Life Sciences, Waltham, MA, USA) in a total volume of 25 μL of reaction mixture containing 12.5 μL of AmpliTaq Gold master mix (Roche, Basel, Switzerland), 50 pmol of each primer and 2 μL of extracted DNA. The PCR reaction was 95°C for 15 minutes, followed by 30 cycles with 95°C for 30 seconds, 56°C for 40 seconds, 72°C for 30 seconds and a final extension step of 72°C for 5 minutes. PCR products were analysed by agarose gel electrophoresis using 2% (w/v) agarose in Tris-EDTA buffer.

SCC*mec* typing was performed by PCR assays as described previously [24], [25]. spa typing was performed as described by Harmsen *et al*. [26]. Clonal relatedness of the spa types was determined by the BURP (Based Upon Repeat Pattern) algorithm as described by Mellmann *et al*. [27]. MLST was performed on all 37 isolates as described by Enright *et al*. [7].

### Detection of genes for antibiotic resistance and virulence

DNA microarray analysis using Identibac *S. aureus* Genotyping Kit 2 (Alere Technology, Jena, Germany) was used following protocols provided by the manufacturer. Data generated were analysed using the ArrayMate software and the ArrayMate Reader (Alere Technology), which assigned MRSA isolates to STs and clonal complexes (CCs) by comparing each isolate to STs and CCs of previously characterized isolates in the ArrayMate database [28], [29].

## Results

The MRSA isolates were obtained from nasal swabs (*n* = 18), skin and soft tissues (*n* = 10), sputum (*n* = 3), groin (*n* = 2), ear (*n* = 2), and vaginal swab and axilla (*n* = 1 each). The patients consisted of 23 men and 13 women. The sex of one patient was not specified.

All 37 isolates were urease negative and were susceptible to vancomycin and teicoplanin (minimum inhibitory concentration ≤1.5 mg/L), linezolid, chloramphenicol, rifampicin, mupirocin and fusidic acid but resistant to trimethoprim (*n* = 28; 75.6%), ciprofloxacin (*n* = 11; 29.7%), erythromycin and clindamycin (*n* = 9; 24.3%), gentamicin and kanamycin (*n* = 8; 21.6%), and tetracycline (*n* = 7; 19.0%).

### Molecular typing of MRSA isolates

**Fig. 1:**
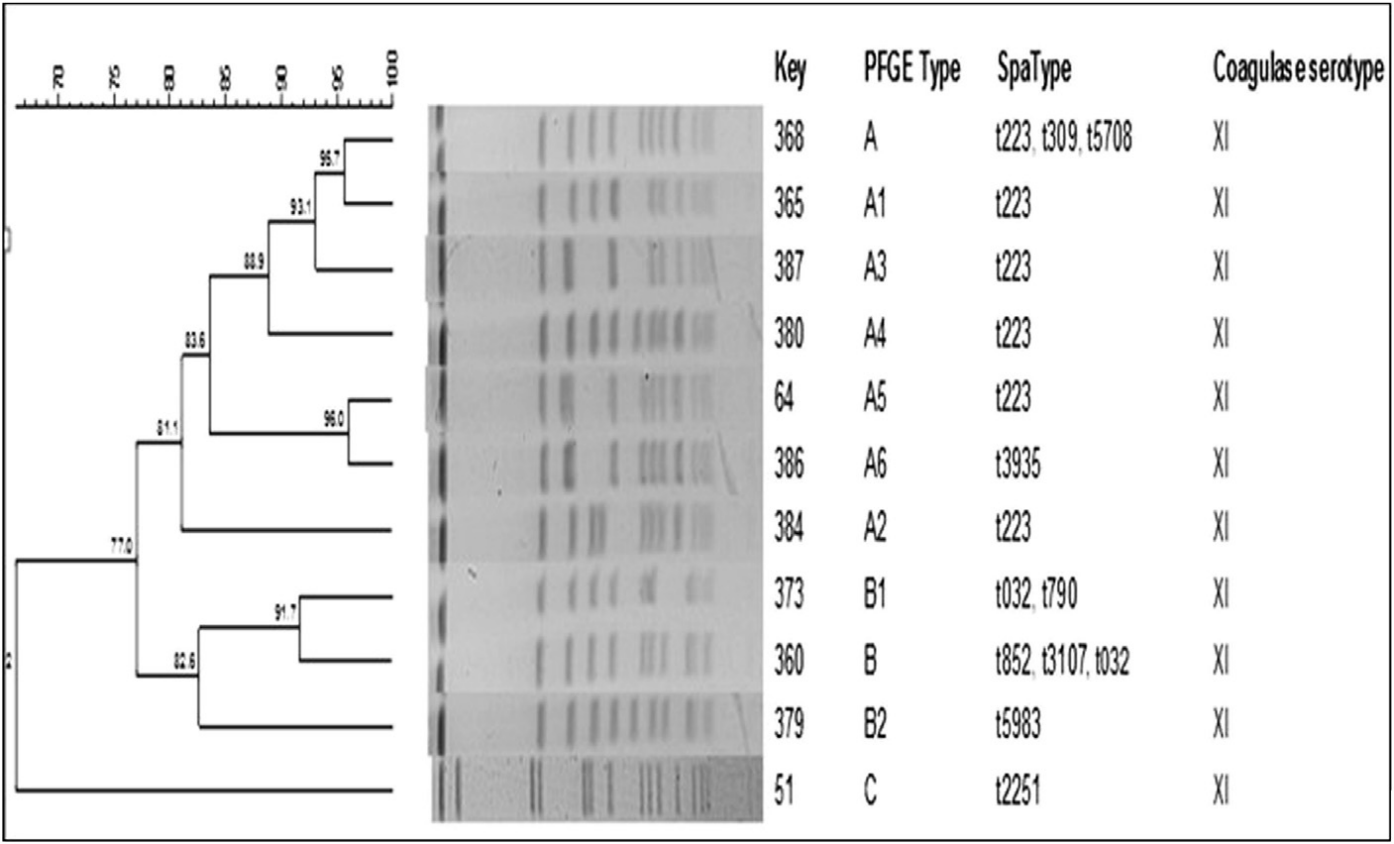
Dendogram of pulsed-field gel electrophoresis patterns of ST22-IV-MRSA isolates. MRSA, methicillin-resistant *Staphylococcus aureus*.

The isolates were grouped into three PFGE patterns, designated types A (22 isolates), B (14 isolates) and C (one isolate), as summarized in Table 1 and Fig. 1.

**Fig. 2:**
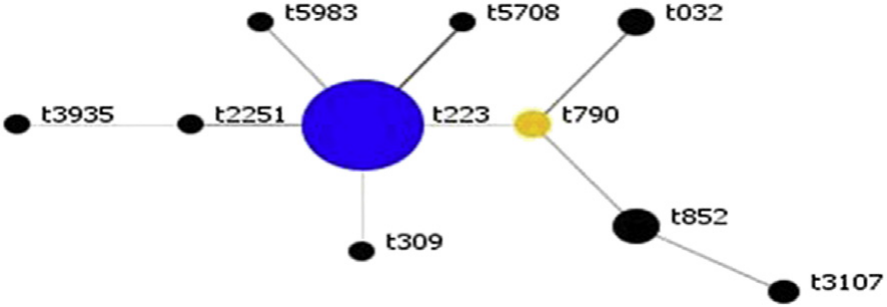
Population snapshot for MRSA BURP analysis. BURP grouping using default parameters resulted in one spa CC. Each dot represents unique spa type. Diameter of dot is proportional to quantity of corresponding spa type. Blue dots represent putative founder (i.e. spa type with highest score within CC); yellow dots, subfounder with second highest score. BURP, Based Upon Repeat Pattern); CC, clonal complex; MRSA, methicillin-resistant *Staphylococcus aureus*.

All 37 isolates belonged to coagulase type XI, ST22, and carried SCC*mec* IV genetic element. SCC*mec* subtyping distinguished the isolates into subtypes IVa (*n* = 26, 70.2%) and IVh (*n* = 3, 8.1%). Eight isolates (21.6%) had no subtypes. The isolates belonged to ten spa types with 19 isolates (51.3%) assigned to spa type t223, followed by t852 (*n* = 5; 13.5%), t790 (*n* = 3; 8.1%), t032 (*n* = 3; 8.1%) and t3107 (*n* = 2; 5.4%). spa types t5983, t309, t5708, t3935 and t2251 occurred in one isolate each. BURP analysis defined one spa CC (spa-CC) 223 with the founder as t223 (Fig. 2).

A combination of the molecular typing results revealed that the isolates were ST22-IVa-t223 (*n* = 19; 51.3%), ST22-IV-t852 (*n* = 5; 13.5%), ST22-IVa-t790 (*n* = 3; 8.1%), ST22-IVh-t032 (*n* = 3; 8.1%) and ST22-IV-t3107 (*n* = 2; 5.4%). ST22-IVa-t309, ST22-IVa-t2251, ST22-IVa-t3935, ST22-IVa-t5708 and ST22-IV-t5983 occurred in one isolate each (Table 2).

### DNA microarray analysis of isolates

DNA microarray analysis revealed that the 37 isolates were positive for genes encoding accessory gene regulator type 1 (*agrI*), capsular polysaccharide type 5 (*cap5*), staphylococcal enterotoxin egc gene cluster (*seg, sei, selm, seln, selo* and *selu*), haemolysin beta (*hlb*), putative membrane protein (*hl*), staphylokinase (*saK*) and chemotaxis inhibition protein (*chp*) but differed in the carriage of *sea, seb, sec, tst* (toxic shock syndrome toxin), *hlgA* (haemolysin gamma A) and *hla* (haemolysin alpha). Eight isolates (21.6%) consisting of t852, t3107 and t5983 were positive for Panton-Valentine leukocidin (PVL) (Table 2). None of the isolates was positive for *sed.*

As shown in Table 2, DNA microarray analysis classified the isolates into three groups: CC22-UK EMRSA-15/Barnim-MRSA clone, CC22-[tst1^+^] UK EMRSA-15/Middle Eastern variant and CC22-PVL^+^ UK EMRSA-15. The CC22-UK EMRSA-15/Barnim-MRSA clone comprised six isolates, which belonged to SCC*mec*-IVh-t032 (three isolates) and SCC*mec* IVa-t790 (three isolates), that were resistant to erythromycin and clindamycin and carried *ermC.* The t790 isolates were positive for *sec* but lacked *hla* and *hlgA,* which were present in t032 isolates.

The CC22-[tst1^+^] UK EMRSA-15/Middle Eastern variant consisted of 23 isolates belonging to spa type t223 (*n* = 19) and one isolate each of t309, t2251, t3935 and t5708. These isolates harboured *tst* and were resistant to trimethoprim but differed in their resistance to tetracycline, erythromycin and clindamycin and carriage of virulence genes.

The CC22-PVL^+^ UK EMRSA-15 variant consisted of eight isolates that belonged to spa types t852 (*n* = 5), t3107 (*n* = 2) and t5983 (*n* = 1). These isolates were PVL positive and were resistant to gentamicin, kanamycin, tobramycin, erythromycin, clindamycin, trimethoprim and ciprofloxacin. The t852 isolates were negative for *scn, hla* and *hlgA.*

## Discussion

The results of this study provide insight into the epidemiology of EMRSA-15 isolates in Kuwait hospitals. Similar to EMRSA-15 isolates reported elsewhere [6], [7], [29], the isolates investigated in this study belonged to ST22 and carried a type IV SCC*mec* genetic element. However, molecular subtyping and DNA microarray analysis revealed differences in their genetic backgrounds, suggesting multiple origins for EMRSA-15 in Kuwait hospitals.

The study revealed that isolates fitting the CC22-IV[tst1^+^] UK EMRSA-15/Middle Eastern variant, consisting of spa types t223 (51.3%), t309 (2.7%), t2251 (2.7%), t3935 (2.7%) and t5708 (2.7%), constituted the dominant EMRSA-15 variant in Kuwait hospitals in 2010. In addition, the t223 isolates widespread in the country evidenced by their isolation in six of the ten hospitals studied. The t223 isolates had antibiotic resistance and virulence profiles similar to PVL-negative, *tst*-positive ST22-IV-t223 isolates reported to have colonized children and parents in the Gaza Strip [30], [31] as well as PVL-negative, *tst*-positive ST22-IV-MRSA-t223 recovered from healthy individuals in Jordan [32], making it the dominant EMRSA-15 clade in the Middle East.

The multiresistant CC22-MRSA-IV-PVL^+^ variant, consisting of spa types t852 (13.5%), t3107 (5.4%) and t5983 (2.7%), was the second most common EMRSA-15 variant in this study. ST22-IV-MRSA-t852 isolates were reported among healthy carriers and patients in Indian hospitals as early as 2008 [13], [33], which may represent the origin of this EMRSA-15 variant. Since then, PVL-positive, multiresistant spa type t852 isolates have been reported among ST22-IV MRSA isolates in Saudi Arabia [17], Qatar [19] and Oman [20], suggesting an increasing transmission of this variant in the Gulf Cooperative Council countries. The t852 isolates were also reported among of ST22-IV MRSA isolated in Zurich, Switzerland, between 2012 and 2014 [34], pointing to their spread in European hospitals.

Surprisingly, the ST22-IVh-t032 isolates related to the UK EMRSA-15/Barnim MRSA clone [10] were less common in this study. Similarly, ST22-IV MRSA related to UK EMRSA-15/Barnim MRSA clone was detected only in 8.9% of MRSA in a hospital in Riyadh, Saudi Arabia [17]. Also, t032, which was the dominant spa type of ST22-IV-MRSA in a United Arab Emirates hospital in 2003, was replaced by t005 in 2008, with none of the 2008 isolates carrying t032 [18]. Furthermore, none of ST22-IV-MRSA reported recently in Qatar [19] and Oman [20] belonged to t032, suggesting a displacement of t032 isolates in Kuwait and other GCC hospitals, in contrast to its continued spread in Europe [8], [10], [29], Malaysia [15] and Singapore [12]. Similar to t032, t790 isolates, which were detected in small numbers in this study, constituted the dominant EMRSA-15 spa type in central Iran [35]. These studies highlight the genetic diversity of ST22-IV subtypes in different countries and the importance of molecular subtyping in understanding their epidemiology.

The UK EMRSA-15/Barnim clone has been associated with invasive infections and has been the dominant cause of bloodstream infections in European countries [3], [8]. In contrast, the ST22 isolates in this study were obtained mostly from skin and soft tissue infections and colonization sites. Similarly, the majority of ST22-IV isolates reported from Indian hospitals [33] and day care centres in the United States [36] were from carriers, probably reflecting the genetic diversity observed in this study and the ability of the clones to survive and proliferate under different environments which support their global spread.

All ST22-IV-MRSA isolates in this study possessed egc (*seg, sei, selm, seln, selo* and *selu*), as has been reported in other studies [29], [37], [38], implying that egc is a major virulence factor for ST22-IV-MRSA isolates. However, the PVL-positive t852 isolates lacked genes for staphylococcal complement inhibitor (*scn*), alpha haemolysin (*hla*) and the A component of haemolysins gamma (*hlgA*), highlighting genetic changes in the emerging variant.

The majority of the ST22-IV-MRSA isolates were resistant to trimethoprim, erythromycin and clindamycin, as has been reported in ST22-IV-MRSA isolates obtained in Ireland [39].

## Conclusions

This study revealed that CC22-IV[tst1^+^] UK EMRSA-15/Middle Eastern variant is the dominant EMRSA-15 variant in Kuwait, followed by the PVL-positive, multiresistant t852 variant, with only few of the isolates related to the European EMRSA-15/Barnim variant of spa type t032. The presence of a mixed population of MRSA isolates poses unique problems for infection control. The study has enriched our understanding of the epidemiology of EMRSA-15 in Kuwait, emphasizing the need for continuous surveillance of MRSA in healthcare facilities to detect changes in their clonal composition and distribution.

## Figures and Tables

**Table 1:** Source and characteristics of MRSA isolates in Kuwait hospitals

Strain No.	Year	Hospital	Gender	Source	PFGE	SCC*mec* type	spa type
Kuwait_70	2005	Armed Forces	M	Sputum	B1	IVh	t032
Kuwait_64	2005	Mubarak	M	Swab	A5	IVa	t223
Kuwait_74	2005	Mubarak	M	Wound	A3	IVa	t223
Kuwait_51	2005	Mubarak	M	Nasal	C	IVa	t2251
Kuwait_270	2010	Farwaniya	M	Nasal	B	IVh	t032
Kuwait_376	2010	CDH	M	Wound	B1	IVh	t032
Kuwait_359	2010	Sabah	F	Nasal	A	IVa	t223
Kuwait_365	2010	Maternity	F	Nasal	A1	IVa	t223
Kuwait_366	2010	Al-Razi	M	Wound	A1	IVa	t223
Kuwait_368	2010	Mubarak	M	Nasal	A	IVa	t223
Kuwait_369	2010	Sabah	M	Nasal	A	IVa	t223
Kuwait_370	2010	Ibn-Sina	M	Nasal	A	IVa	t223
Kuwait_371	2010	Al-Razi	F	Nasal	A	IVa	t223
Kuwait_372	2010	Jahra	F	Ear	A	IVa	t223
Kuwait_378	2010	Mubarak	M	Nasal	A	IVa	t223
Kuwait_380	2010	Maternity	—	Nasal	A4	IVa	t223
Kuwait_384	2010	Ibn-Sina	M	Nasal	A2	IVa	t223
Kuwait_385	2010	Ibn-Sina	M	Axilla	A2	IVa	t223
Kuwait_387	2010	Mubarak	F	Nasal	A3	IVa	t223
Kuwait_388	2010	Mubarak	M	Bed sore	A	IVa	t223
Kuwait_391	2010	Sabah	M	Nasal	A2	IVa	t223
Kuwait_398	2010	Mubarak	M	Nasal	A1	IVa	t223
Kuwait_400	2010	Sabah	F	Ear	A	IVa	t223
Kuwait_229	2010	Maternity	F	Nasal	A	IVa	t309
Kuwait_361	2010	Ibn-Sina	M	Swab	B	IV	t3107
Kuwait_363	2010	Sabah	M	Sputum	B	IV	t3107
Kuwait_386	2010	Al-Razi	M	Ulcer	A6	IVa	t3935
Kuwait_377	2010	Sabah	M	Groin	A	IVa	t5708
Kuwait_379	2010	Adan	M	Nasal	B2	IV	t5983
Kuwait_373	2010	Maternity	F	HVS	B1	IVa	t790
Kuwait_374	2010	Sabah	M	Nasal	B1	IVa	t790
Kuwait_375	2010	Ibn-Sina	F	Sputum	B1	IVa	t790
Kuwait_360	2010	Adan	F	Abscess	B	IV	t852
Kuwait_362	2010	Farwaniya	F	Nasal	B	IV	t852
Kuwait_364	2010	Mubarak	M	Groin	B	IV	t852
Kuwait_367	2010	Adan	F	Pus	B	IV	t852
Kuwait_399	2010	Maternity	F	Pus	B	IV	t852

CDH, Chest Disease hospital; MRSA, methicillin-resistant *Staphylococcus aureus;* HVS, high vaginal swab; PFGE, pulsed-field gel electrophoresis.

**Table 2:** Molecular characteristics of ST22 MRSA isolates

Year	No. of isolates	Strain definition	spa type	Antibiotic resistance (*n*)	Antibiotic resistance genes	Toxins encoding genes	Miscellaneous genes
**UK EMRSA-15/Barnim**							
2005	1	ST22-MRSA-IVh	t032	E, CC, CIP	*ermC*	*seb, sec sel,* egc	scn, hla, hlIII, hlgA
2010	2	ST22-MRSA-IVh	t032	E (1), CC (1), CIP (2), W(1)	*ermC*	egc	scn, hla, hlgA
2010	3	ST22-MRSA-IVa	t790	E (2), CC (2)	*ermC*	*sec,* egc	scn
**UK EMRSA-15/Middle Eastern variant [tst**^**+**^**]**							
2005	2	ST22-MRSA-IVa	t223	E (1), CC (1), TE (2), W (2)	*ermC, tetK, dfrS1*	*tst,* egc	scn, hla, hlgA
2010	17	ST22-MRSA-IVa	t223	TE (4), W(16)	*tetK, dfrS1*	*tst,* egc	scn, hla, hlgA
2010	1	ST22-MRSA-IVa	t309	W	*dfrS*1	*tst,* egc	scn, hla, hlgA
2005	1	ST22-MRSA-IVa	t2251	TE	*tetK*	*tst,* egc	scn, hla, hlgA
2010	1	ST22-MRSA-IVa	t3935	W	*dfrS1*	*tst, sea* egc	scn, hla
2010	1	ST22-MRSA-IVa	t5708	W	*tetK, dfrS1*	*tst,* egc	scn
**CC22-MRSA-IV-PVL**^**+**^							
2010	5	ST22-MRSA-IV	t852	CN (5), K (5), E (1), CC (1), TOB (4), W (3), CIP (5)	*aacA-aphD, aadD*	PVL, egc	
2010	2	ST22-MRSA-IV	t3107	CN (2), K (2), TOB (1), E (2), CC (2), W (2), CIP (2)	*ermC, aacA-aphD, aadD, dfrS1*	PVL, egc	scn, hla, hlgA
2010	1	ST22-MRSA-IV	t5983	E, CC, W, CIP, CN, K, TOB	*ermC, aacA-aphD, aadD, dfrS1*	PV, egc	scn, hla

All isolates carried hemolysin delta (hld) egc gene cluster (*seg, sei, selm, seln, selo, selu*).

CC, clindamycin; chp, chemotaxis-inhibiting protein; CIP, ciprofloxacin; CN, gentamicin; E, erythromycin; hl, putative membrane protein; hla, haemolysin alpha; hlb, haemolysin beta; hlgA, haemolysin gamma, component A; hlIII, putative membrane protein; K, kanamycin; MRSA, methicillin-resistant *Staphylococcus aureus*; PVL, Panton Valentine leukocidin; sak, staphylokinase; scn, staphylococcal complement inhibitor; TE, tetracycline; TOB, tobramycin; tst, toxic shock syndrome toxin; W, trimethoprim.
